# Prediction of tannin content and quality parameters in astringent persimmons from visible and near-infrared spectroscopy

**DOI:** 10.3389/fpls.2023.1260644

**Published:** 2023-12-18

**Authors:** Min Woo Baek, Han Ryul Choi, In Geun Hwang, Shimeles Tilahun, Cheon Soon Jeong

**Affiliations:** ^1^ Department of Horticulture, Kangwon National University, Chuncheon, Republic of Korea; ^2^ Interdisciplinary Program in Smart Agriculture, Kangwon National University, Chuncheon, Republic of Korea; ^3^ National Institute of Horticultural and Herbal Science, Rural Development Administration, Wanju-gun, Republic of Korea; ^4^ Frusen Co., LTD, Yongin, Republic of Korea; ^5^ Agriculture and Life Science Research Institute, Kangwon National University, Chuncheon, Republic of Korea; ^6^ Department of Horticulture and Plant Sciences, Jimma University, Jimma, Ethiopia

**Keywords:** persimmon, astringency, tannin, non-destructive PLS model, Vis/NIR spectra

## Abstract

**Introduction:**

Tannin content and postharvest quality characteristics of persimmon fruit are often determined by the destructive analysis that consumes time, does not allow the acquisition of data from the same fruit continuously, and requires expensive high-performance equipment. This research was done to investigate the potential for non-destructive estimation of astringency and quality parameters in persimmon fruit based on visible/near-infrared (VNIR) spectra.

**Methods:**

VNIR spectra readings, the reference tannin content, and quality parameters were measured from fruits of “Cheongdo-Bansi” and “Daebong” persimmon cultivars at harvest and throughout the ripening/deastringency period. The spectra readings from half of the total fruit were utilized for the calibration set, while the other half readings were used for the prediction set. To develop models correlating the spectra data to the measured reference parameters data, the partial least square regression (PLSR) method was utilized.

**Results and discussion:**

In the case of ‘Daebong’, the coefficients of determination (R^2^) between VNIR spectra and the actual measured values of TSS, firmness, simple sugars, and tannin content were (0.95, 0.94, 0.96, and 0.96) and (0.93, 0.89, 0.96, and 0.93), for the calibration and prediction sets, respectively. Similarly, the R^2^-values of (0.86, 0.93, 0.79, and 0.81) and (0.83, 0.91, 0.75, and 0.75) were recorded in ‘Cheongdo-Bansi’ for the calibration and prediction sets, respectively. Additionally, the acquired data were divided into two sets in a 3:1 ratio to develop predictive models and to validate the models in multiple regressions. PLSR models were developed in multiple regression to estimate the tannin content of both cultivars from firmness and simple sugars with R^2^-values of 0.83 and 0.79 in ‘Cheongdo-Bansi’ for the calibration and prediction sets, respectively, whereas, R^2^-values of 0.80 and 0.84 were recorded in ‘Daebong’ for the calibration and prediction sets, respectively. The overall findings of this study showed the possibility of using VNIR spectra for the prediction of postharvest quality and tannin contents from intact persimmon fruit with quick, chemical-free, and low-cost assessment methods. Also, the multiple regression using physicochemical parameters could fairly predict the tannin content in persimmon fruit though destructively but save time and low-cost.

## Graphical Abstract

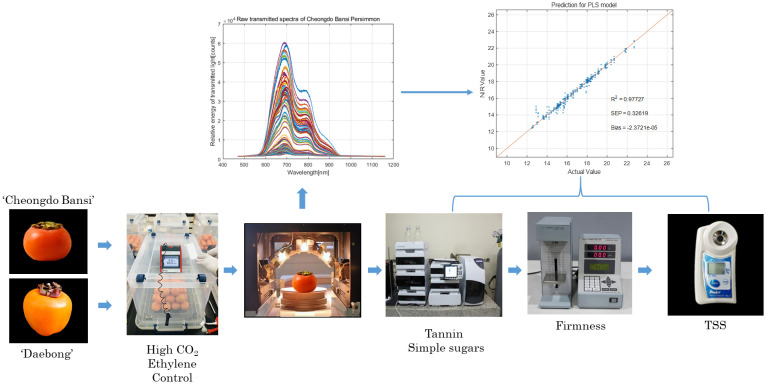

## 1 Introduction

Persimmon (*Diospyros kaki* Thunb.) probably originated in China and has been mainly grown in China, Korea, and Japan as a relevant food source from prehistoric times (Parfitt et al., 2015). In 2021, China, the Republic of Korea, and Japan contributed 96% of the world’s persimmon production (FAOSTAT, 2021). According to FAOSTAT (2021), persimmon production in the Republic of Korea was 200,610 tons from the total Asia and world production of 4.16 and 4.33 million tons, respectively. Persimmon fruit classifies as either astringent or non-astringent, and it is a delicious and healthy fruit rich in vitamins, minerals, and antioxidants which are associated with numerous health benefits (Park et al., 2017; Das and Eun, 2021). ‘Cheongdo-Bansi’ and ‘Daebong’ are commercially important astringent persimmon cultivars that are commonly grown in the Republic of Korea due to their adaptability to the environment and excellent taste and texture (Park et al., 2017; Park et al., 2019). Tannins are polyphenol compounds with a high molecular weight that cause astringency due to their large hydroxyl phenolic groups (Cortés et al., 2017). The soluble tannins gradually turn into insoluble tannins as the fruit ripens and the fruit become less astringent (Noypitak et al., 2015; Cortés et al., 2017). In non-astringent persimmon, soluble tannin is reduced naturally during ripening, while in astringent persimmon, a high level of soluble tannin is maintained when it is not fully ripe and fruits cannot be eaten during the commercial harvest stage because of their higher levels of soluble tannins (Yamada et al., 2002; Akagi et al., 2009; Das and Eun, 2021). Fruits of both ‘Cheongdo-Bansi’ and ‘Daebong’ cultivars, however, undergo rapid softening after harvest, and by the time astringency is low enough to be palatable, the fruits become too soft. Conversely, firm textured fruits which are suitable for distribution are astringent. This astringency can cause a dry or puckering sensation in the mouth that can be unpleasant (Das and Eun, 2021). Hence, it requires rapid ripening or removing the astringency from persimmons for agreeable palatability.

However, many studies were reported to achieve fast removal of astringency from persimmon including ethylene (Park et al., 2017; Park et al., 2019), high concentrations of CO_2_ (Arnal and Del Río, 2003; Salvador et al., 2007), ethanol (Ortiz et al., 2005), high (Ben-Arie and Sonego, 1993) and freezing (Das and Eun, 2021) temperatures treatments. Treatments with ethylene and high concentration of CO_2_ (high CO_2_) are the most widely used commercial techniques that promote fast ripening and astringency removal, respectively (Cortés et al., 2017; Park et al., 2017). Ethylene treatment causes rapid expression of ripening-related genes (Park et al., 2017; Park et al., 2019), and exposing the fruit to a high CO_2_ promotes the accumulation of acetaldehyde due to anaerobic respiration in the fruit. The soluble tannins become insoluble as they react with the acetaldehyde and the astringency is thus eliminated (Cortés et al., 2017).

Firmness is the main difference between the persimmon fruit deastringed by the treatment with ethylene and the high CO_2_. Fruit treated with ethylene becomes softer, and acquires a jelly-like consistency which is difficult to distribute. Yet, some consumers prefer the taste and store it in a deep freezer for future use after fully ripens. On the other hand, the firm texture of the fruit is maintained during deastringency with the high CO_2_, appreciated by the industry and consumers in its suitability for distribution (Munera et al., 2017a). Therefore, optimum ripening and astringency removal are required to avoid loss of fruit quality caused by high concentration or long treatment and residual astringency due to low concentration or short treatment of ethylene or high CO_2_ (Arnal et al., 2008; Novillo et al., 2014; Park et al., 2017). Hence, it is important to measure tannin contents during the treatment periods to ensure optimum ripening and deastringency.

The common methods used to measure the changes in tannin contents during ripening and astringency removal are usually destructive and thus the same fruit cannot be monitored continuously. The analysis also requires expensive high-performance equipment and consumes time. Therefore, having a reliable, low-cost, fast, and easy-to-implement method for tannin determination in persimmons is a useful tool for astringency management during postharvest handling and distribution. Predictive models developed by using visible and near-infrared (VNIR) spectroscopy and color variables are among the most common techniques currently used for the prediction of secondary metabolites such as lycopene and β-carotene in tomatoes and α-solanine and α-chaconine in potatoes (Tilahun et al., 2018; Tilahun et al., 2020). The interaction between VNIR range spectra and the organic molecules that make up a compound helps to obtain qualitative and quantitative information from the spectra (Pasquini, 2003; Tilahun et al., 2020).

Cortés et al. (2016) predicted the internal quality (combination of total soluble solids (TSS), firmness, and flesh color) of mango with VNIR reflection spectroscopy. Noypitak et al. (2015) also developed PLSR models to evaluate tannin content in astringent ‘Xichu’ persimmon and recommended NIR interactance spectroscopy for optimal prediction of soluble tannin content. In addition, Cortés et al. (2017) also reported the possibility of determining astringency through reflectance VNIR spectra at selected points in intact and half-cut ‘Rojo Brillante’ persimmon fruit. Similarly, Son et al. (2009) predicted sugar contents in a sweet persimmon using reflectance spectra. Most of the previous works if not all, used CO_2_ treatment to remove the astringency of persimmon during nondestructive estimations. However, more samples at different levels of astringency and softening are needed to ascertain the prediction power of astringency and ripening quality to fulfill the demands of both consumers and the industry. Thus, this study included the treatment with ethylene or high CO_2_ and untreated control of the intact ‘Daebong’ and ‘Cheongdo-Bansi’ persimmon fruits. This work determines the possibility of nondestructive estimation of astringency and quality parameters including TSS, firmness, and simple sugars by using VNIR spectroscopy in transmittance mode, in combination with a multivariate analysis technique, to predict the changes in quality and tannin content of persimmon fruits during ripening and deastringency.

## 2 Materials and methods

### 2.1 Plant material, treatments and storage at ambient condition

**Figure 1:**
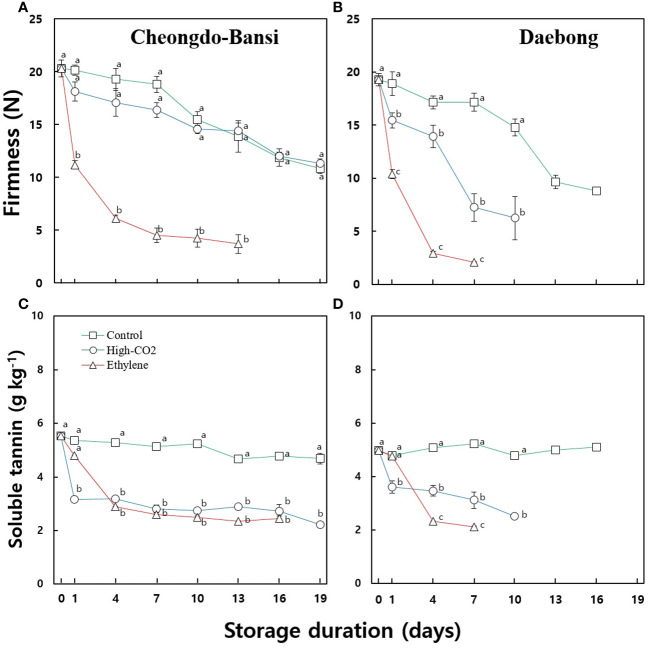
Changes in firmness and soluble tannin content of the control and ethylene or high CO_2_ treated ‘Cheongdo-Bansi’ **(A, C)** and ‘Daebong’ **(B, D)** persimmon fruit during storage at 22°C. Each data point indicates 5-10 fruits. The number of fruit at each measurement day was started with five fruits at the beginning of the storage and decided afterward to 5-10 fruits for each measurement based on the fruit status.

Astringent persimmon fruits (*Diospyros kaki* Thunb. ‘Cheongdo-Bansi’ and ‘Daebong’) were harvested from Jeollanamdo, Yeoungham, Korea on 28 Sept. 2022. After harvest, 150 uniform fruits free of external damage were selected from each cultivar. Within 12 hours of harvest, the fruits were then brought to the postharvest laboratory at the Department of Horticultural Sciences, Kangwon National University, Korea. After keeping at ambient condition for 3 hours to remove field heat, uniform fruits free of defects were carefully reselected and divided into three groups (control, ethylene treatment, and high CO_2_ treatment, 40 fruits each) for both cultivars. So, 120 fruits of each cultivar were used for the experiment. The treatment groups were treated separately with 100 mg kg^-1^ ethylene (Park et al., 2017) and 95% CO_2_ (Arnal et al., 2008) for 24 h in a sealed 62 L container at 22°C. The control fruits were treated under similar conditions without ethylene and CO_2_ treatment. The fruits were characterized as 117.8 ± 1.96 and 271.5 ± 1.73 g of fresh weight, 20.29 ± 0.8 and 19.26 ± 0.9 N of firmness, 16.93 ± 0.5 and 17.48 ± 0.4% of TSS, and 4.68 ± 0.2 and 4.98 ± 0.3 g kg^-1^ of soluble tannin at harvest for ‘Cheongdo-Bansi’ and ‘Daebong’, respectively. To include different levels of astringency, data for the destructively collected parameters (tannin content, firmness, TSS, and simple sugars) and spectroscopic measurements of the intact fruits were acquired at harvest, on the first day after harvest, and at 3-day intervals afterward until the fruit attain the end of their shelf life (**Figure 1**). The number of fruits at each measurement day was started with five fruits at the beginning of the storage and decided afterward to 5-10 fruits based on the fruit status.

### 2.2 VNIR spectral acquisition and analysis

**Figure 2:**
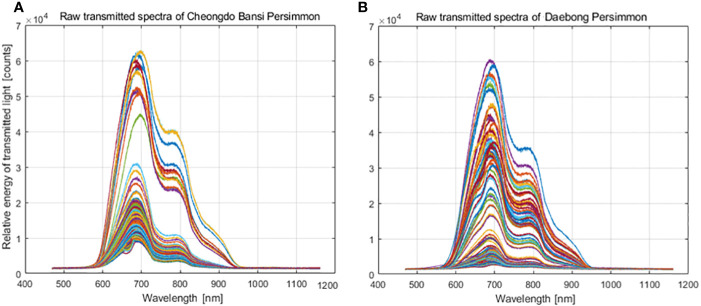
Transmittance energy spectra curves obtained from and ‘Cheongdo-Bansi’ **(A)** and ‘Daebong’ **(B)** persimmon fruit by using VNIR spectrometer.

In accordance with Tilahun et al. (2020; Tilahun et al., 2018), each individual intact fruit of the ‘Cheongdo-Bansi’ and ‘Daebong’ cultivars were scanned in the transmittance mode in the spectral region of 500-1100 nm using three (12 V/100 W) halogen lamps as a source of VNIR light. Fruit holder was used to keeping the fruit right above the detector (**Figure 1B**). The integration time was set to 100 ms and the measurement was done 12 times from different directions. The fruit was placed on the fruit holder to prevent it from falling, and the fruit holder was rotated above the detector to avoid the interference of scanning by human hand (4 positions on stem-end plane, middle plane, and stylar-end plane (Noypitak et al., 2015) per fruit) to introduce variability within the fruit samples. For each measurement, a total of 3500 data points was captured at 0.2 nm sampling intervals. The VNIR spectrometer was linked to a computer to transfer data. A total of 1440 spectra readings from 120 fruits for ‘Cheongdo-Bansi’ and 1440 spectra readings from 120 fruits for ‘Daebong’ were acquired from the intact persimmon fruit throughout the ripening/deastringency period. After removing outliers, a total of 1200 spectra readings (10 readings per fruit) were chosen for analysis from each ‘Cheongdo-Bansi’ and ‘Daebong’ (**Figure 2**). For each cultivar, the spectra readings from half of the total fruit (600 readings from 60 fruits) were utilized for the calibration set, while the other half readings (600 readings from 60 fruits) were used for the prediction set using the leave one sample out procedure to separate the sample sets. The original spectra were transformed by multiplicative scattering correction (MSC), first derivatives, the Hanning window, and standard normal variate (SNV) to remove undesired information and reduce systematic noise. The prediction was based on the lowest predicted residual error sum of squares (PRESS) value, which was used to determine the ideal number of latent variables for the partial least squares regression (PLSR) model. To determine a linear relationship between measured references and spectral data, MATLAB R2012b (Version 8.0.0.783, The Math Works, Inc., Natick, MA, USA) was used to conduct PLSR regression analysis. RMSEC (root mean square of standard error in calibration), RMSEP (root mean square of standard error in prediction), coefficient of determination for calibration (Rc^2^) and prediction (Rp^2^) were used to evaluate the performance of the developed PLSR models. A predictive model with higher Rp^2^, small bias values and lower RMSEP is considered as a reliable prediction model.

### 2.3 Measurements of fruit quality parameters and analysis

The measurements for firmness, TSS, soluble tannin content, fructose and glucose content were made from each whole fruit according to the methodology implemented in our laboratory and described by Park et al. (2017). A Rheo meter (Sun Scientific Co. Ltd., Tokyo, Japan) with a 10 kgf maximum force of penetration and a 3 mm round, flat-ended stainless-steel probe was utilized to measure the firmness of the intact persimmon fruit with a probe speed of 1 mm/s around equatorial area of each fruit. TSS was measured by utilizing a digital refractometer (Atago Co. Ld., Tokyo, Japan) and 5 g of homogenized persimmon pulp juice from each whole fruit. Glucose and fructose contents were measured in accordance with the method employed by Park et al. (2016); 5 g of each whole fruit’s frozen pulp sample was added to 50 mL of distilled water, homogenized, and then the juice was centrifuged (Mega-17R, Hanil Science Industrial, Korea) at 12,578 × g for 10 min and the supernatant was filtered through 0.45 μm membrane filter (Advantec, Tokyo, Japan). The analysis was carried out using HPLC with a RI detector (Waters 410 Differential Refractometer, Waters, MA, USA) and a Sugar-Pak ™ column (6.5 × 300 mm, Waters, USA) with an injection volume of 10 μL. Soluble tannin content was measured with the modified Folin-Dennis method (Park et al., 2017). Samples of 5 g from whole fruit were added directly into a solution of 25 mL of 80% methanol. Then, 6 mL of distilled water was added to 1 mL of filtered supernatant sample solution. The mixture was then vortexed after 0.25 mL of 2 N Folin-Ciacalteau reagent had been added. Saturated Na_2_CO_3_ (1 mL) and distilled water (1.5 mL) were added after 3 min. Following a 1 h incubation period at 25°C, the absorbance of mixed sample was measured with a spectrometer (Thermo Fisher Scientific, Waltham, MA, USA) at 725 nm, and the results were reported as g kg^−1^ on a fresh weight basis.

To perform PLSR models using the above spectra readings (10 readings per fruit) obtained from different directions of a fruit, measured data for reference parameters (tannin, firmness, SSC, glucose, and fructose) were collected from a total of 240 fruit samples. These samples comprised 120 fruit from each of ‘Cheongdo-Bansi’ and ‘Daebong’, with 8 replications per fruit sample, and the mean value of each 4 replicates was used as the fifth value for each parameter to make 10 replicates per fruit to get a one to one fit with the spectra readings.

To examine the effectiveness of multivariate regression models to estimate tannin content (astringency), the values of the collected parameters were divided into calibration and prediction sets using the leave one sample out procedure to separate the four sample sets. Fruit quality parameters were collected from both cultivars throughout the storage period, and the data were divided in to 3:1 ratio. 80 fruit samples were used for calibration, and 40 fruit samples were used for prediction. A total of 240 fruit samples (‘Cheongdo-Bansi’ and ‘Daebong’, 120 each) were used for the experiment in 8 replications per fruit sample and the mean value was calculated for analysis. The measured reference parameters (tannin, firmness, SSC, glucose and fructose) were organized in excel, where the rows represented the number of samples (the total of 120 averaged value from 120 persimmons for each cultivar), and the columns represented the number of variables (X and Y variables). The X-variables, or predictors, were the values of measured firmness, SSC, glucose and fructose values associated with each sample. The Y-variables, or response, were the measured tannin values associated with each sample. Multivariate PLSR models were developed from calibration data set and the measured reference data of each parameter were compared to the predicted data obtained by PLSR models in both the calibration and prediction data sets. A predictive model with higher Rp^2^, lower RMSEP, and higher ratio of prediction to deviation (RPD) is thought to be a good prediction model. RPD is calculated by the ratio of SD to RMSEC/P, where SD is the standard deviation of the observed parameters. If the RPD value is less than 1.5, the calibration is not usable. When the RPD is between 1.5 and 2.0, it becomes able to distinguish between high and low values, but when it is between 2.0 and 2.5, it becomes possible to make approximate quantitative predictions (Cortés et al., 2016).

## 3 Results

### 31 Firmness and soluble tannin content of persimmon during ripening/deastringency

**Figure 1** shows the changes in firmness and soluble tannin contents of the control and ethylene or high CO_2_ treated ‘Cheongdo-Bansi’ and ‘Daebong’ persimmon fruit during storage at 22°C. The firmness and soluble tannin data showed significant differences between the treatments starting from the first day. Ethylene treated fruits ripened faster, became softer, and acquired a jelly-like consistency which reduced their storage life up to 13 and 7 d for ‘Cheongdo-Bansi’ and ‘Daebong’, respectively, compared to the controls that reached 19 and 16 d for ‘Cheongdo-Bansi’ and ‘Daebong’, respectively. Conversely, high CO_2_ treatment hastened deastringency and maintained firmness and stored up to 19 and 10 d for ‘Cheongdo-Bansi’ and ‘Daebong’, respectively. Ethylene treatment hastens softening in both cultivars while high CO_2_ maintained firmness and prolonged the storability of both cultivars, and its effect was distinctly higher in the case of ‘Cheongdo-Bansi’ (**Figure 1**).

### 32 VNIR spectra vs. reference analysis

**Figure 3:**
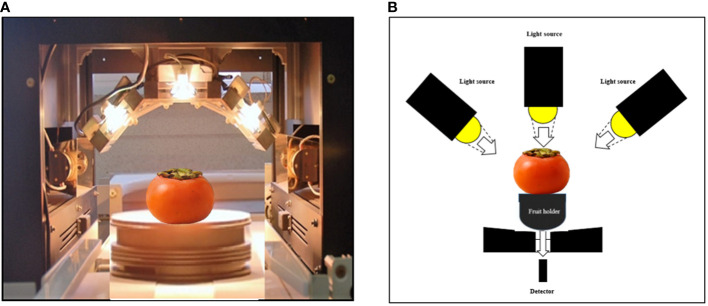
VNIR spectrometer **(A)** and measurement system **(B)** during transmittance spectra measurement of intact persimmon fruits.

In this study, transmittance energy spectra of ‘Cheongdo-Bansi’ and ‘Daebong’ intact astringent persimmon fruit were recorded by VNIR spectrometer (**Figures 3A, B**) in the wavelength of 500-1000 nm as shown in **Figures 2A, B**. As indicated in **Figure 2**, differences were observed in the raw transmitted spectra characteristics of the two cultivars. More scattered spectra were observed in ‘Cheongdo-Bansi’ than ‘Daebong’.

**Figure 4:**
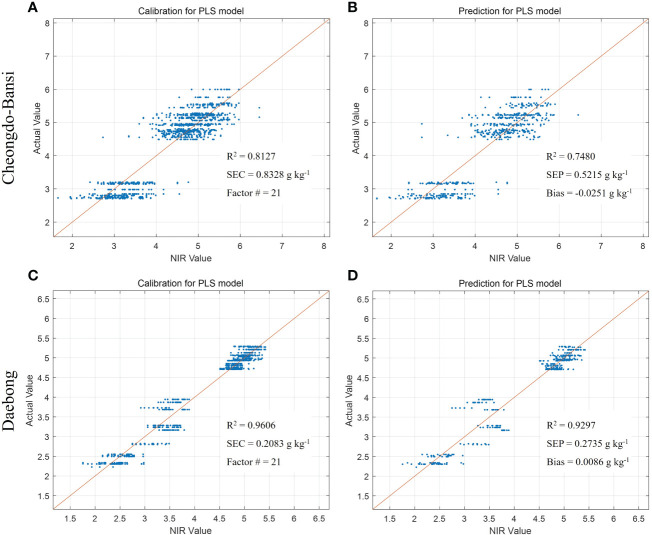
Measured vs. predicted values of tannin content (g kg^-1^) in ‘Cheongdo-Bansi’ for calibration **(A)** and prediction **(B)**, and ‘Daebong’ for calibration **(C)** and prediction **(D)** sets with PLS models. The scatter plot depicts the prediction accuracy of the model. The x axis represents the predicted value of tannin content and the y axis represents the measured value by the PLS models.

In addition to the PLSR models for the estimation of tannin contents to determine astringency levels of the two persimmon cultivars, PLSR models were also developed to predict postharvest quality parameters such as firmness, TSS, and simple sugars (glucose and fructose). **Table 1** shows the essential data for VNIR modeling and multiple regressions. Promising results were recorded for both cultivars with higher predictive models in ‘Daebong’ than ‘Cheongdo-Bansi’. In ‘Cheongdo-Bansi’, Rc^2^ and RMSEC for measured vs. VNIR values of tannin in the calibration set were 0.81 and 0.83 g kg^-1^, respectively. Similarly, Rp^2^ and RMSEP for measured vs. VNIR values of tannin in the prediction set were 0.75 and 0.52 g kg^-1^, respectively (**Figures 4A, B**). On the other hand, in ‘Daebong’, Rc^2^ and RMSEC for measured vs. VNIR values of tannin in the calibration set were 0.96 and 0.21 g kg^-1^, respectively, while Rp^2^ and RMSEP for measured vs. VNIR values of tannin in the prediction set were 0.93 and 0.27 g kg^-1^, respectively (**Figures 4C, D**).

**Table 1:** Firmness, TSS, simple sugars and soluble tannin content data used for VNIR modeling and multiple regressions.

Cultivars	Parameters	Treatments	Storage duration (days)							
			0	1	4	7	10	13	16	19
Cheongdo-Bansi	Firmness (N)	Control	20.29 ± 0.79	20.14 ± 0.47	19.27 ± 1.02	18.79 ± 0.79	15.48 ± 0.72	13.88 ± 1.49	11.87 ± 0.84	10.83 ± 0.45
		High-CO_2_	20.29 ± 0.79	18.12 ± 0.90	17.08 ± 1.29	16.38 ± 0.68	14.54 ± 0.42	14.41 ± 0.75	12.02 ± 0.33	11.34 ± 0.39
		Ethylene	20.29 ± 0.79	11.18 ± 0.39	6.10 ± 0.35	4.5 ± 0.69	4.24 ± 0.87	3.68 ± 0.87	–	–
	TSS (%)	Control	16.93 ± 1.12	17.15 ± 0.79	17.48 ± 0.45	19.41 ± 0.37	19.43 ± 0.24	18.55 ± 0.34	20.04 ± 0.42	19.39 ± 0.56
		High-CO_2_	16.93 ± 1.12	15.25 ± 0.43	15.02 ± 0.15	15.83 ± 0.59	16.69 ± 0.18	16.31 ± 0.34	17.10 ± 0.51	18.30 ± 0.70
		Ethylene	16.93 ± 1.12	15.55 ± 0.29	16.90 ± 0.32	16.98 ± 0.55	17.96 ± 0.36	20.11 ± 0.86	21.08 ± 0.63	–
	Glucose (mg kg^-1^)	Control	5822.23	6269.41	6030.09	7558.36	7833.15	7969.14	8618.81	8760.82
		High-CO_2_	5822.23	5202.74	5585.59	6070.89	6297.67	6564.06	6630.25	6404.56
		Ethylene	5822.23	5808.23	6048.38	6044.46	6534.75	6678.41	6448.98	–
	Fructose (mg kg^-1^)	Control	5865.22	6178.98	5891.16	7146.67	7328.13	7461.81	7944.08	7986.70
		High-CO_2_	5865.22	4502.56	4751.30	5146.50	5366.50	5523.44	5357.71	5139.43
		Ethylene	5865.22	5300.90	5506.17	5412.71	6110.06	6303.45	5996.22	–
	Soluble tannin (g kg^-1^)	Control	4.68 ± 0.09	4.69 ± 0.03	5.53 ± 0.01	5.23 ± 0.01	4.78 ± 0.07	5.28 ± 0.09	5.14 ± 0.01	5.37 ± 0.21
		High-CO_2_	4.68 ± 0.09	3.16 ± 0.07	2.75 ± 0.05	1.80 ± 0.15	3.19 ± 0.01	2.89 ± 0.04	2.72 ± 0.26	2.21 ± 0.01
		Ethylene	4.68 ± 0.09	4.80 ± 0.01	5.17 ± 0.00	4.88 ± 0.01	4.59 ± 0.04	3.82 ± 0.01	3.53 ± 0.09	–
Daebong	Firmness (N)	Control	19.26 ± 0.59	18.89 ± 1.11	17.13 ± 0.60	17.15 ± 0.86	14.79 ± 0.80	9.65 ± 0.62	8.79 ± 0.17	–
		High-CO_2_	19.26 ± 0.59	15.44 ± 0.71	13.90 ± 1.06	7.24 ± 1.31	6.23 ± 2.04	–	–	–
		Ethylene	19.26 ± 0.59	10.37 ± 0.41	2.92 ± 0.17	2.05 ± 0.06	–	–	–	–
	TSS (%)	Control	17.48 ± 0.73	18.60 ± 0.41	18.25 ± 0.40	19.76 ± 0.59	18.61 ± 2.20	17.31 ± 0.89	15.20 ± 1.01	–
		High-CO_2_	17.48 ± 0.73	15.69 ± 0.28	14.65 ± 0.41	16.40 ± 0.39	15.89 ± 0.45	–	–	–
		Ethylene	17.48 ± 0.73	15.48 ± 0.59	14.81 ± 0.57	16.03 ± 0.33	–	–	–	–
	Glucose (mg kg^-1^)	Control	4933.13	5108.14	5006.48	5371.05	5858.40	5499.64	5861.58	–
		High-CO_2_	4933.13	4979.20	4224.52	5210.59	5369.60	–	–	–
		Ethylene	4933.13	4523.83	5006.09	5515.52	–	–	–	–
	Fructose (mg kg^-1^)	Control	4032.12	4162.41	4097.25	4353.54	4727.80	4453.36	4751.39	–
		High-CO_2_	4032.12	3788.34	3242.84	3817.81	4010.23	–	–	–
		Ethylene	4032.12	3558.48	3919.92	4174.34	–	–	–	–
	Soluble tannin (g kg^-1^)	Control	4.97 ± 0.03	4.79 ± 0.02	5.09 ± 0.02	5.23 ± 0.02	4.79 ± 0.07	5.00 ± 0.03	5.10 ± 0.10	–
		High-CO_2_	4.97 ± 0.03	3.60 ± 0.23	3.46 ± 0.20	3.12 ± 0.31	2.52 ± 0.02	–	–	–
		Ethylene	4.97 ± 0.03	4.76 ± 0.05	2.32 ± 0.01	2.12 ± 0.03	–	–	–	–

Ethylene treated fruits ripened faster, became softer, and acquired a jelly-like consistency which reduced their storage life up to 13 and 7 d for ‘Cheongdo-Bansi’ and ‘Daebong’, respectively. The number of fruits at each measurement day was started with five fruits at the beginning of the storage and decided afterward to 5-10 fruits for each measurement based on the fruit status.

**Figure 5:**
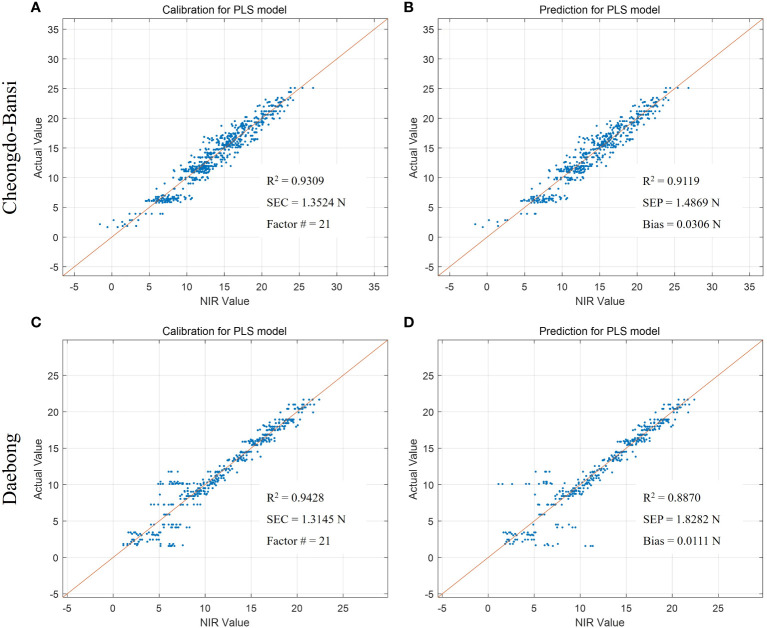
Measured vs. predicted values of firmness (N) in ‘Cheongdo-Bansi’ for calibration **(A)** and prediction **(B)**, and ‘Daebong’ for calibration **(C)** and prediction **(D)** sets with PLS models. The scatter plot depicts the prediction accuracy of the model. The x axis represents the predicted values of firmness and the y axis represents the measured value by the PLS models.

**Figure 6:**
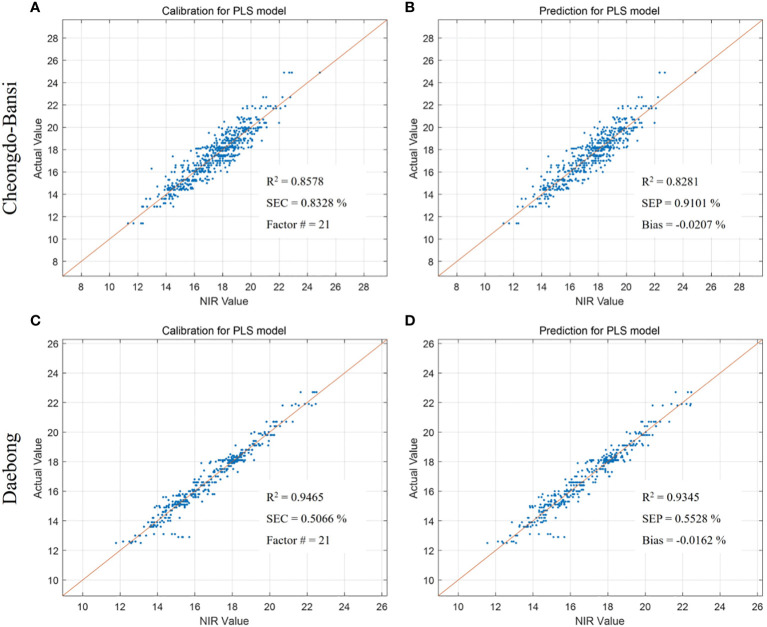
Measured vs. predicted values of TSS (%) in ‘Cheongdo-Bansi’ for calibration **(A)** and prediction **(B)**, and ‘Daebong’ for calibration **(C)** and prediction **(D)** sets with PLS models. The scatter plot depicts the prediction accuracy of the model. The x axis represents the predicted value of TSS and the y axis represents the measured value by the PLS models.

**Figure 7:**
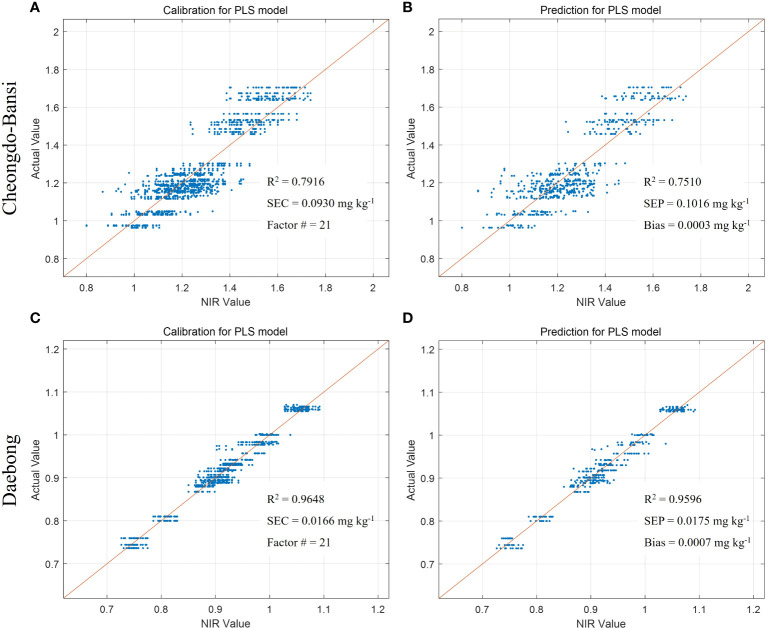
Measured vs. predicted values of simple sugars (fructose and glucose (mg kg^-1^)) in ‘Cheongdo-Bansi’ for calibration **(A)** and prediction **(B)**, and ‘Daebong’ for calibration **(C)** and prediction **(D)** sets with PLS models. The scatter plot depicts the prediction accuracy of the model. The x axis represents the predicted value of simple sugars and the y axis represents the measured value by the PLS models.

The same trends of higher predictive models were also observed in ‘Daebong’ than ‘Cheongdo-Bansi’ for measured vs. VNIR values of firmness, TSS and simple sugars. In case of firmness, Rc^2^ and RMSEC were 0.94 and 1.31 N, and 0.93 and 1.35 N in ‘Daebong’ and ‘Cheongdo-Bansi’, respectively (**Figures 5A, C**). Correspondingly, Rp^2^ and RMSEP were 0.89 and 1.83 N, and 0.91 and 1.49 N for ‘Daebong’ and ‘Cheongdo-Bansi’, respectively (**Figures 5B, D**). Rc^2^ and RMSEC for measured vs. VNIR values of TSS were 0.95 and 0.51%, and 0.86 and 0.83%, whereas Rp^2^ and RMSEP were 0.93 and 0.55%, and 0.83 and 0.91% in ‘Daebong’ and ‘Cheongdo-Bansi’, respectively (**Figures 6A–D**). Regarding the simple sugars (glucose and fructose), higher predictive models of 0.96 and 0.02 mg kg^-1^, and 0.96 and 0.02 mg kg^-1^ for Rc^2^ and RMSEC, and Rp^2^ and RMSEP, respectively, were observed in ‘Daebong’ (**Figures 7C, D**). In ‘Cheongdo-Bansi’, Rc^2^ and RMSEC, and Rp^2^ and RMSEP were 0.79 and 0.09 mg kg^-1^, and 0.75 and 0.10 mg kg^-1^, respectively (**Figures 7A, B**).

### 33 Multivariate PLSR models using the reference data

**Table 2** shows the means and ranges of reference (measured) tannin content in the calibration and prediction data sets that acquired by the destructive analysis. Meanwhile, tannin content that estimated by multivariate PLSR model using firmness, glucose and fructose in the calibration and prediction data sets are also presented in **Table 2**. For ‘Cheongdo-Bansi’, Rc^2^, RMSEC and RPD values of the calibration data set were 0.83, 0.27 g kg^-1^ and 0.36, respectively. In the prediction data set, the corresponding values were 0.79, 0.42 g kg^-1^ and 0.22, respectively for Rp^2^, RMSEP and RPD (**Table 2**). Similarly, Rc^2^, RMSEC and RPD values of the calibration data set were 0.79, 0.50 g kg^-1^ and 0.38, respectively for ‘Daebong’. The corresponding values were 0.84, 0.53 g kg^-1^ and 0.43, respectively for Rp^2^, RMSEP and RPD in the prediction data set (**Table 2**).

**Table 2:** Statistics for multivariate calibration and prediction of tannin content in ‘Cheongdo-Bansi’ and ‘Daebong’ persimmon fruit.

Cultivar	Set	Parameters	Fruit number	Total samples	Mean	Range	SD	Rc/p^2^	RMSEC/P	RPD
Cheongdo-Bansi	Calibration	Reference tannin content	80	640	4.49	2.80-5.44	0.84			
		Multivariate			4.38	2.35-5.45	0.78	0.83	0.36	2.3
	Prediction	Reference tannin content	40	320	4.19	2.73-5.44	1.07			
		Multivariate			4.24	2.58-5.44	0.94	0.79	0.49	2.2
Daebong	Calibration	Reference tannin content	80	640	4.10	2.30-5.29	1.05			
		Multivariate			3.95	2.20-5.40	0.96	0.79	0.50	2.1
	Prediction	Reference tannin content	40	320	4.03	2.30-5.29	1.20			
		Multivariate			4.20	2.42-5.37	0.97	0.84	0.53	2.3

SD, standard deviation; RMSEC, root mean square error of calibration; RMSEP, root mean square error of prediction; RPD, residual prediction deviation (SD/RMSEC/P); Rc^2^, coefficient of determination in calibration; Rp^2^, coefficient of determination in prediction data set.

Following the predictive analysis in multiple regression, firmness, glucose and fructose values were found to have high predictive *p*-values in the prediction of tannin from the measured reference postharvest quality parameters. The following equations were found to be the best equations.


$$\begin{array}{c} Tannin\;\left(g\;kg^{-1}\right)\;=3.36-0.06\;\left(firmness\right)\\ -19.67\left(fructose\right)\;+24.54\left(glucose\right)-\mathrm{‘Cheongdo-Bansi’} \end{array}$$

$$Tannin\;\left(g\;kg^{-1}\right)\;=2.1\;+\;0.06\;\left(firmness\right)-35.21\left(fructose\right)\;+47.74\left(glucose\right)-\mathrm{‘Daebong’}$$

**Figure 8:**
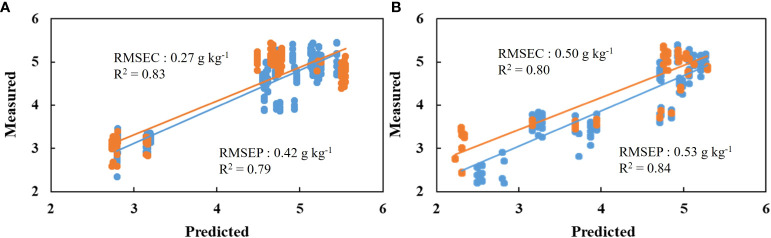
Measured vs. predicted scores of tannin content (g kg^-1^) for ‘Cheongdo-Bansi’ **(A)** and ‘Daebong’ **(B)** in the calibration (blue) and prediction (red) sets with multivariate PLS models using firmness and simple sugars values. The scatter plot depicts the prediction accuracy of the model. The x axis represents the predicted value of tannin content and the y axis represents the measured value by the multivariate PLS models.

The calibration and prediction set with multivariate PLSR models had shown encouraging results to utilize the models based on the measured reference vs. predicted scores of both cultivars. For the prediction data set, a multivariate PLSR model had the highest coefficient of correlation (0.84) for ‘Daebong’ and (0.79) for ‘Cheongdo-Bansi’ (**Table 2**; **Figure 8**).

## 4 Discussion

Rapid ripening, deastringency, and softening in ethylene treated persimmon fruit could be due to rapid expression of ripening-related genes (Park et al., 2017; Park et al., 2019). On the other hand, the deastringency of firm persimmon fruit by high CO_2_ treatment could be due to the accumulation of acetaldehyde in the fruit by anaerobic respiration, and the soluble tannins become insoluble as they react with the acetaldehyde (Cortés et al., 2017). Firmer fruit in high CO_2_ treated fruit can be explained by the reduction of respiration rate which in turn inhibit the effect of internal ripening hormone, ethylene (Tilahun et al., 2022).

The introduction of environmentally friendly nondestructive technology like VNIR spectroscopy, which has achieved widespread recognition for assessing food quality, is necessary to meet the present demand for high-quality products (Tilahun et al., 2020). More scattered spectra in ‘Cheongdo-Bansi’ than ‘Daebong’ could be attributed to the relatively more sample variation in ‘Cheongdo-Bansi’ during the extended storage period up to 16, 19, and 19 days for ethylene, high CO_2_, and control, respectively. In contrast, ‘Daebong’ had a shorter storage period of only 7, 10, and 16 days for ethylene, high CO_2_, and control, respectively.

From the results of this study, the performance of PLSR models for the prediction of tannin content in intact persimmon fruit was cultivar dependent. Noypitak et al. (2015) evaluated tannin content in high CO_2_ treated and control intact ‘Xichu’ persimmon fruits using NIR and reported PLSR models with 0.94 and 0.95 Rp^2^ in transmittance and interactance modes, respectively. They suggested to use reflected light than transmitted light due to the variation of soluble tannin content in the flesh close to the skin and at the core. Cortés et al. (2017) also reported PLSR models using the data obtained from high CO_2_ treated intact fruits of ‘Rojo Brillante’ persimmon at six measurement points in reflectance mode with Rp^2^ of 0.90 and 0.91 with all and selected wavelengths, respectively. Nevertheless, our results of this study revealed the possible application of VNIR spectra in transmittance mode to predict tannin content in intact persimmon fruit with higher predictive models in ‘Daebong’ than ‘Cheongdo-Bansi’. Previous studies by Tilahun et al. (2020, Tilahun et al., 2018) on tomatoes and potatoes also support the use of spectra in transmittance mode to predict lycopene, β-carotene, and glycoalkaloids. Moreover, the novelty of this study lies in its incorporation of both deastringency and ripening treatments, encompassing persimmon fruits exhibiting varying degrees of astringency and firmness.

Munera et al. (2017b) reported the potential of hyperspectral imaging to predict firmness with Rp^2^ of 0.80 in ‘Rojo Brillante’ persimmon fruit. Cortés et al. (2016) also predicted internal quality (combination of TSS, firmness and flesh color) of mango with VNIR reflection spectroscopy and reported Rp^2^ between 0.83–0.88 using full spectral range. Similarly, Ar et al. (2019) demonstrated the possibility of using NIR spectroscopy to predict TSS and firmness with Rp^2^ of 0.86 and 0.94, respectively, in astringent ‘Rendeu’ persimmon fruit, while there was low accuracy in predicting vitamin C and total acid due to their low contents in persimmon. It is important to note that, in addition to estimating astringency, the developed PLSR models in the current work can be used as the better nondestructive tools for the assessment of the firmness and TSS in both cultivars. Similar to our present study, Liu et al. (2006) reported best models for the prediction of simple sugars in intact apples using Fourier transform near-infrared (FT-NIR) spectroscopy. Taken together, the feasibility of using VNIR to predict all dependent variables (quality parameters and astringency level in terms of tannin content) of persimmon fruit were indicated by lower RMSEC/P values and higher Rp^2^ between 0.89-0.96 and 0.75-0.91 for ‘Daebong’ and ‘Cheongdo-Bansi’, respectively. The wide range of NIR values in the developed PLSR models could be due to ten spectra readings obtained from different directions from one fruit, whereas eight reference measured values were collected per fruit. In addition, the variation in the nature of the astringency treatments (control, high CO_2_, and ethylene) has led to variations in fruit characteristics. Notably, tannin content decreased in both high CO_2_ and ethylene treatments, contributing to a narrower range of actual tannin content values.

In our previous works, the multivariate PLSR models were developed to predict lycopene and β-carotene in tomatoes and glycoalkaloids in potatoes from Hunter’s color values (Tilahun et al., 2018; Tilahun et al., 2020). Measurements of postharvest quality parameters such as color values, firmness, TSS, and simple sugars (glucose and fructose) were taken during the experiment. However, in this study, the PLSR models for color values and TSS in the calibration data set had lower R^2^ and the *p*-values were higher than 0.15 for both ‘Cheongdo-Bansi’ and ‘Daebong’. Hence, color values and TSS were not included in multivariate PLSR model development. Instead, we included simple sugars (glucose and fructose) data for PLSR model development. The above indicated multivariate PLSR models could not be claimed as nondestructive estimation method as they utilize the destructively acquired data for model development. However, astringency levels can be estimated from firmness and simple sugars without the extra analysis of tannin content. This in turn, reduce time, cost of skilled man power and solvents, and does not require expensive high-performance equipment. As the present study incorporated only two cultivars, further studies are needed on various cultivars to develop more robust multivariate PLSR models.

## 5 Conclusions

The present study indicates attempts to predict tannin content and quality parameters in intact persimmon fruit with chemical-free, fast and cheap VNIR spectra. Multivariate PLSR models were also developed from the reference measured parameters including firmness, TSS, and simple sugars. Prediction of tannin content, firmness, TSS, and simple sugars was promising in both cultivars, and relatively better predictive models were developed in ‘Daebong’ than ‘Cheongdo-Bansi’ with both VNIR and multivariate-based techniques. Our models could be promising alternative tools to the costly and time-consuming destructive analysis. The developed models could benefit both the industry and consumers through their use in the agricultural processing and distribution centers to sort fruits on a conveyor belt at different levels of astringency and ripening stages with a VNIR spectrometer. In addition, astringency levels can be estimated from firmness and simple sugars by the developed multivariate PLSR models without the extra analysis of tannin content. Further investigation on different cultivars at different levels of astringency and softening to evaluate tannin content and ripening quality of intact persimmon fruit could help to develop more robust models.

## Data availability statement

The original contributions presented in the study are included in the article/supplementary material. Further inquiries can be directed to the corresponding authors.

## Author contributions

MB: Conceptualization, Formal analysis, Writing – original draft, Writing – review & editing, Data curation, Investigation, Methodology. HC: Formal analysis, Investigation, Methodology, Writing – review & editing. IH: Formal analysis, Methodology, Writing – review & editing, Software, Validation. ST: Formal analysis, Writing – review & editing, Conceptualization, Writing – original draft. CJ: Conceptualization, Funding acquisition, Project administration, Supervision, Writing – review & editing.
